# Novel, alternative splicing signature to detect lymph node metastasis in prostate adenocarcinoma with machine learning

**DOI:** 10.3389/fonc.2022.1084403

**Published:** 2023-01-13

**Authors:** Ping Xie, Jesur Batur, Xin An, Musha Yasen, Xuefeng Fu, Lin Jia, Yun Luo

**Affiliations:** ^1^ Department of Urology, The Third Affiliated Hospital of Sun Yat-Sen University, Guangzhou, Guangdong, China; ^2^ Department of Urology, The First People’s Hospital of Kashi Prefecture, Kashi, Xinjiang, China; ^3^ Department of Urology, The People's Hospital of Suining County, Xuzhou, Jiangsu, China

**Keywords:** alternative splicing (AS), prostate cancer, lymph node metastasis, TCGA, machine learning

## Abstract

**Background:**

The presence of lymph node metastasis leads to a poor prognosis for prostate cancer (Pca). Recently, many studies have indicated that gene signatures may be able to predict the status of lymph nodes. The purpose of this study is to probe and validate a new tool to predict lymph node metastasis (LNM) based on alternative splicing (AS).

**Methods:**

Gene expression profiles and clinical information of prostate adenocarcinoma cohort were retrieved from The Cancer Genome Atlas (TCGA) database, and the corresponding RNA-seq splicing events profiles were obtained from the TCGA SpliceSeq. Limma package was used to identify the differentially expressed alternative splicing (DEAS) events between LNM and non-LNM groups. Eight machine learning classifiers were built to train with stratified five-fold cross-validation. SHAP values was used to explain the model.

**Results:**

333 differentially expressed alternative splicing (DEAS) events were identified. Using correlation filter and the least absolute shrinkage and selection operator (LASSO) method, a 96 AS signature was identified that had favorable discrimination in the training set and validated in the validation set. The linear discriminant analysis (LDA) was the best classifier after 100 iterations of training. The LDA classifier was able to distinguish between LNM and non-LNM with an area under the receiver operating curve of 0.962 ± 0.026 in the training set (D1 = 351) and 0.953 in the validation set (D2 = 62). The decision curve analysis plot proved the clinical application of the AS-based model.

**Conclusion:**

Machine learning combined with AS data could robustly distinguish between LNM and non-LNM in Pca.

## 1 Introduction

Prostate cancer (PCa) is one of the most prevalent malignancies and the second leading cause of death in men in the United States (1). The incidence of prostate cancer, as well as its malignancy, increases with aging (1). About 3% patients have metastases at diagnosis, and the prevalence of metastatic prostate cancer has been climbing, especially among men aged between 55 and 66 (2). Metastasis is most likely to occur in the lymph nodes adjacent to the primary tumor, the pelvic lymph nodes (2). Pelvic lymph node metastasis (LNM) is one of the most decisive factors associated with post-operation disease recurrence and correlates with poor prognosis (3–5). The management of TxcN1M0 prostate cancer is at the crossroads of local and systemic cancer treatment (6). It is crucial to ascertain the status of lymph nodes and accurate lymph node staging will provide patients with better treatment options (7).

By now, there are two major ways to identify the lymph node metastasis before surgery, including imaging modalities and nomograms. Even the advanced imaging modalities, like positron emission tomography/computed tomography (PET/CT) with prostate-specific membrane antigen (PSMA), show moderate sensitivities (50–66%) for LNM detection (8). Nomograms are common clinical predictive models that are based on imaging, pathological, and clinical parameters. The area under the receiver operating characteristic curve (AUC) of three nomograms predicting LNM reported by Partin and Memorial Sloan Kettering Cancer Center (MSKCC), Briganti, ranges from 0.778 to 0.793 (9). Although some progress has been made in predictive models, the performance of these models needs to be improved. However, other than traditional methods, there are few tools to detect lymphatic metastasis in prostate cancer.

In recent years, gene signatures have been reported as a means to predict lymph node metastasis in lung adenocarcinoma and endometrial cancer, as well as prostate cancer (9–11). Alternative splicing (AS), as a specific modality of gene expression, plays a key role in gene expression regulation and gene mutation modulation, and even castration resistance of prostate cancer (10–12). AS is important in carcinogenesis and the immune microenvironment, which affects the prognosis and treatment response in a variety of tumors (10, 13–15).

The association between AS and lymph node metastasis has not been reported previously. The relationship between AS and lymph node metastasis should be elucidated in order to assess the biological behavior of AS in tumors in order to provide individualized optimal treatment to patients.

In this study, we investigated the DEAS events that correlated with LNM and tried to assess the ability of AS features to detect LNM and non-LNM in Pca. We hypothesized that LNM would have a particular AS pattern associated with it when compared to non-LNM, which could distinguish LNM from non-LNM. We identified DEAS events and built an AS-based model with machine learning on a training set and validated its potency on an internal validation set.

## 2 Methods

### 2.1 Data collection

RNA-seq FPKM (Fragments Per Kilobase per Million) profiles and clinical information about the status of lymph nodes of the TCGA prostate adenocarcinoma (PRAD) cohort were acquired from the TCGA data portal (https://portal.gdc.cancer.gov). The corresponding RNA-seq splicing events profiles of PRAD were obtained from the TCGA SpliceSeq (16). In order to get reliable AS event data, we adopted a rigorous screening filter criteria with a sample proportion of PSI values of no less than 75% (17). The cases without the status of lymph node or lack of matched RNA-seq splicing events profiles were excluded. Finally, there were 413 cases included in our analysis cohort.

### 2.2 Identification of differentially expressed alternative splicing events

**Figure 1:**
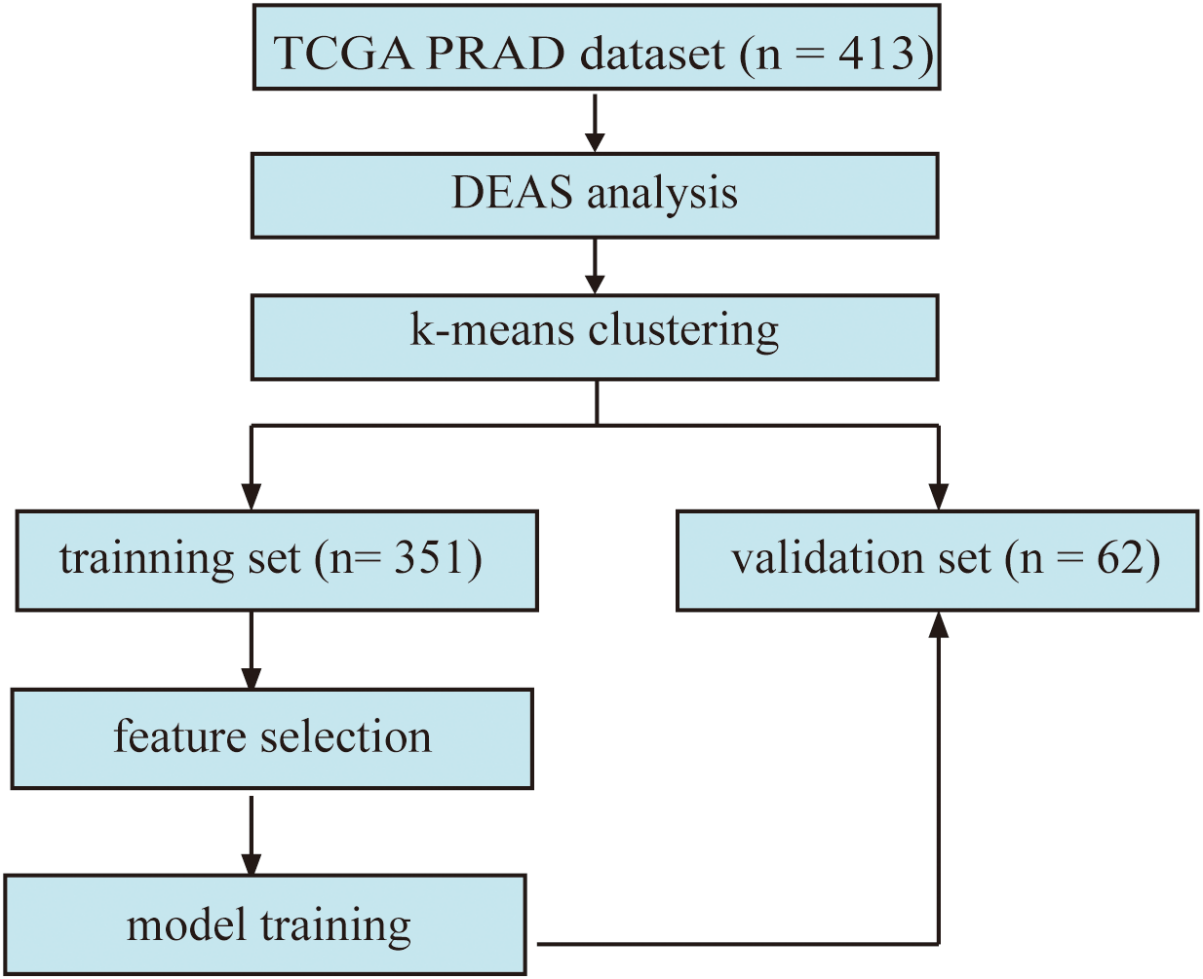
The flowchart displays the framework of our study. (DEAS: differentially expressed alternative splicing).

The overview of the workflow is shown in **Figure 1**. The PRAD cohort was divided into two groups by the presence or absence of lymph node metastasis. We used the limma package (18) for differential analysis. An adjusted p value < 0.05 was applied as the threshold to determine the DEAS.

### 2.3 Feature evaluation

For the purpose of evaluating and analyzing the whole dataset structure. We performed unsupervised clustering on the DEAS feature pool by k-means clustering. Cluster analysis was performed in order to determine LNM patients clustering patterns without knowing the results in advance and evaluated by comparing the clustering outcome with the underlying facts.

### 2.4 Feature selection

The dataset was split randomly into a training set and a validation set, containing 351 cases in the training set and 62 cases in the validation set (**Supplementary Table 1**). To lessen feature redundancy, two feature selection methods were used in the training set to identify features that might be essential for our model. In the first step, a correlation filter premised on the absolute values of pairwise Spearman’s correlation coefficient was applied. The threshold was set at 0.8 for ρ. In a nutshell, if two features have ρ > 0.8, the function examines the mean absolute correlation of each feature, and the feature with the higher mean absolute correlation will be eliminated. In the second step, the least absolute shrinkage and selection operator (LASSO) logistic regression algorithm was applied to choose the most optimized predictive features from the features selected from correlation filter algorithm.

### 2.5 AS-based model construction

In this study, we built eight machine learning classifiers, namely random forest (RF), multi-layer perceptron (MLP), logistic regression (LR), gaussian naive bayes (GNB), linear discriminant analysis (LDA), quadratic discriminant analysis (QDA), support vector machine (SVM), and light gradient boosting machine (LGBM). The eight machine learning classifiers were trained on the training set, respectively. To evaluate the performance and overall error estimation of the eight classifiers, we applied a stratified five-fold cross-validation method with 100 iterations. Oversampling method was not applied in this study, as the ratio of positive samples to negative samples is approximately equal to 1:5. In the training set, each fold in turn was used as a validation set, and the other four folds were used as a training set. The validation outcomes from 100 five-fold cross-validation were then integrated to present a measure of global performance.

### 2.6 Statistical analysis and model explanation

The statistical analysis was performed with the R (version 4.1.2) and the Python (3.9.7) programming languages and environments with the scikit-learn repository. The LGBM classifier was built on the lightgbm module. The performance of the classifiers was assessed by area under the receiver operating characteristic curve (AUC), accuracy, recall/sensitivity, specificity,and F1-score. P value of less than 0.05 indicates statistical significance.

SHAP (SHapley Additive exPlanations) was performed to explain the model. SHAP is a game theoretic method to explain the output of any machine learning model (19). SHAP values evaluate the significance of the output resulting from the inclusion of one feature for all combinations of other features.

## 3 Results

### 3.1 Overview of AS events in the TCGA PRAD cohorts

Finally, there were 413 cases included in our analysis cohort, with 336 lymph node negative cases and 77 lymph node positive cases. 44070 AS events were preliminary identified from 10381 genes, including 3524 AA events in 2488 genes, 3101 AD events in 2185 genes, 9035 AP events in 3621 genes, 8663 AT events in 3781 genes, 16772 ES events in 6578 genes, 228 ME events in 221 genes, 2747 RI events in 1849 genes (**Supplementary Figure 1**).

### 3.2 Identification of differentially expressed AS events associated with lymph node metastases

In this work, 333 important DEAS events in 255 genes were identified by the limma package as being correlated with lymph node metastasis by comparing LNM and non-LNM cases (**Supplementary Table 2**). There were 157 ascending DEAS events and 177 descending events. Compared with the proportion in all AS types, the proportion of AT type in DEAS increased more apparently than any other type, which indicated that AT type may perform a crucial role in tumor metastasis (**Supplementary Figure 1**).

### 3.3 Analysis of the feature set with unsupervised clustering

**Figure 2:**
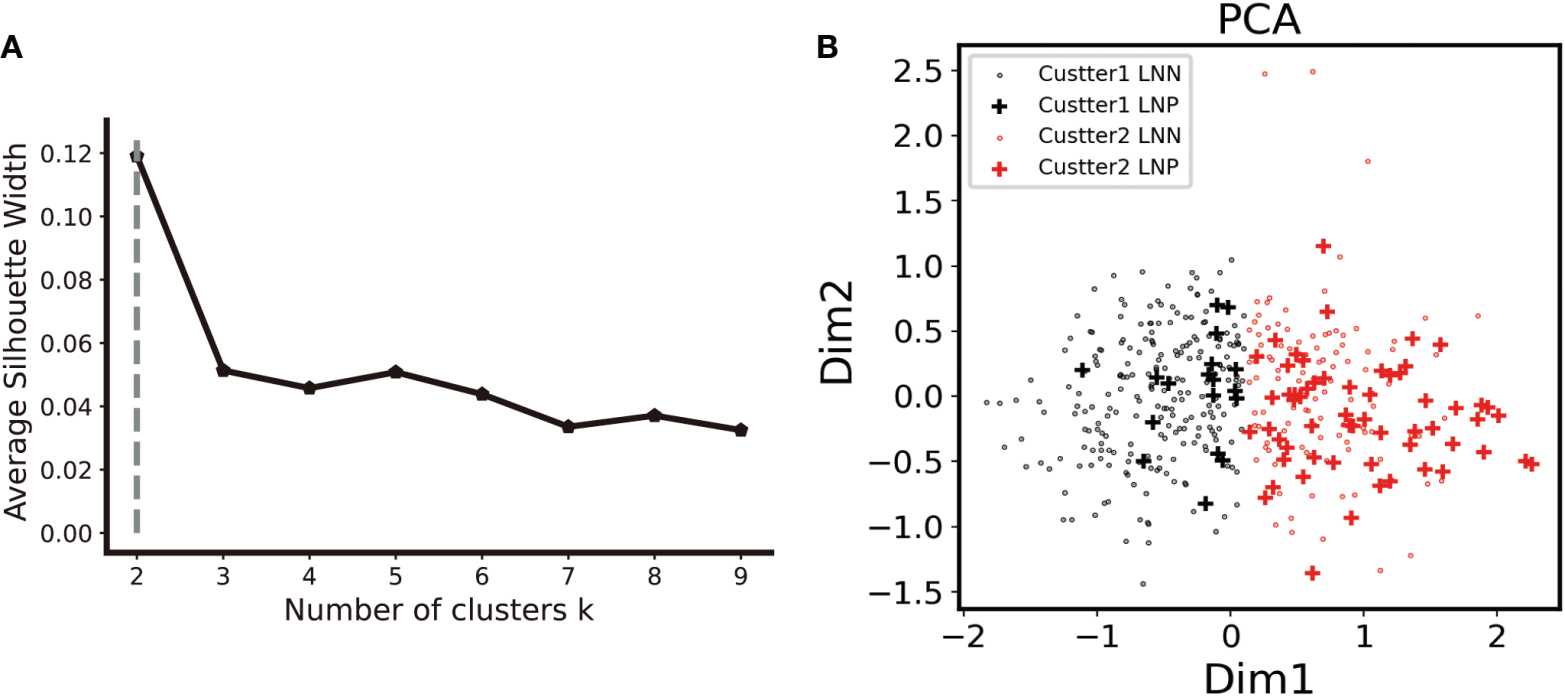
Unsupervised clustering analysis. **(A)**. The elbow curve plot shows that the optimal number of clusters was observed to be two by using K- mean clustering analysis on the PRAD cohort with the 333 DEAS feature. **(B)**. In the two clusters, cluster two had a 76.6% compactness with LNM. (LNN lymph node negative, LNP lymph node positive).

Principal component analysis as well as k-means clustering was used to analyze the whole DEAS feature stream and two clusters were detected. A 76.6% compactness, the degree to which group members share similarities, was detected inside the clusters. The established cluster was validated by applying the silhouette coefficient (silhouette width), which is an algorithm to evaluate the cluster results. The silhouette plot showed that the clustering using two groups was perfect, with no negative silhouette width and the majority of cluster values greater than 0.03, as shown in **Figure 2A**. The lymph node positivity rate was 0.074 in cluster 1 and 0.347 in cluster 2. 76.6% of LNM were collocated in cluster 2 according to the unsupervised clustering algorithm (**Figure 2B**). Most of LNM patients appeared to be clustered together, potentially suggesting that DEAS is a reliable data source to distinguish between LNM and non-LNM.

### 3.4 Features selection

**Figure 3:**
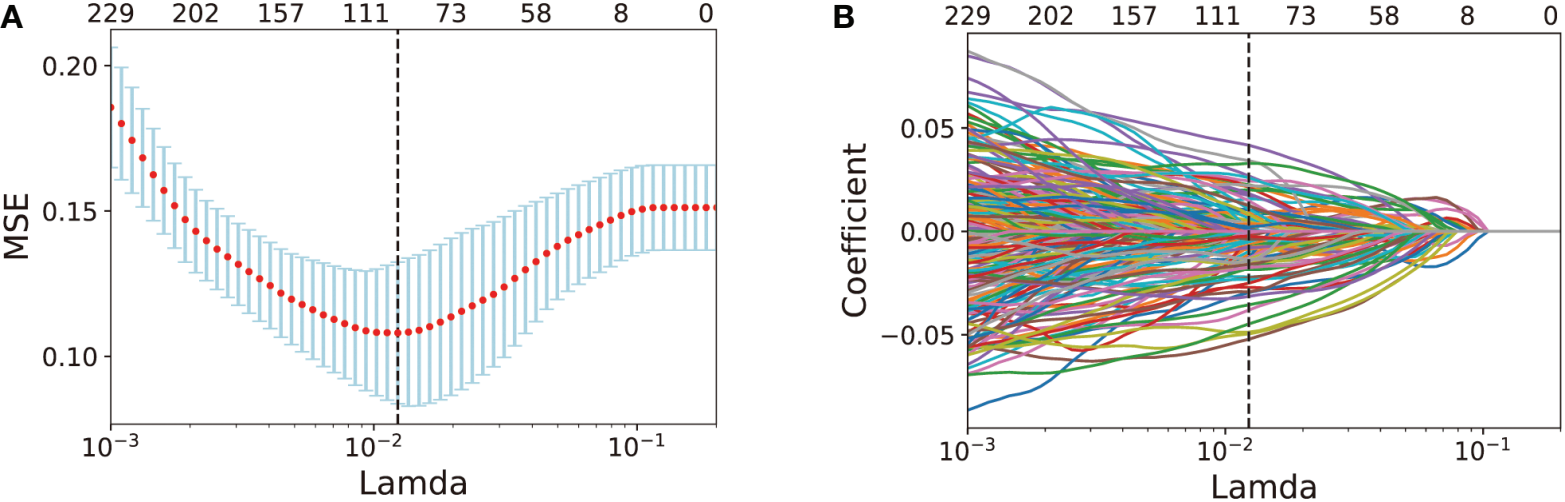
AS feature selection performed by LASSO analysis. **(A)** Selection of the tuning parameter *λ* in the LASSO model *via* 10-fold cross-validation in the training set. optimal *λ* value of 0.0123, with log (*λ*)= -4.396, was selected based on minimum criteria. **(B)**. LASSO coefficient profiles of the 268 DEAS features. Vertical dot line was drawn at the optimal value where optimal λ resulting in 96 nonzero coefficients.

First, 268 key features from 333 DEAS were acquired by using pairwise Spearman’s correlation filter with a 0.8 correlation coefficient threshold in the training set. Second, applying the LASSO-based feature selection method (**Figures 3A, B**), we further selected 96 crucial features from 268 key DEAS (**Supplementary Table 3**).

### 3.5 Supervised classifier performance

The final selected 96 features were then treated as input layers in the subsequent classifiers. With stratified five-fold cross-validation on the training set, we ran 100 iterations on each of the eight distinct classifiers to evaluate their performance. Performance of the eight classifiers was shown in **Supplementary Table 4**. Due to unbalanced data, we selected the optimal classifier based on F1-score and finally the LDA model was chosen with the best F1-score performance. The performance outcome showed that the LDA algorithm achieved an average AUC of 0.962± 0.026 and an accuracy of 0.929± 0.028, a specificity of 0.958± 0.024, a sensitivity of 0.812± 0.111, a F1-socre of 0.809± 0.079 on the stratified five-fold cross-validation set

**Figure 4:**
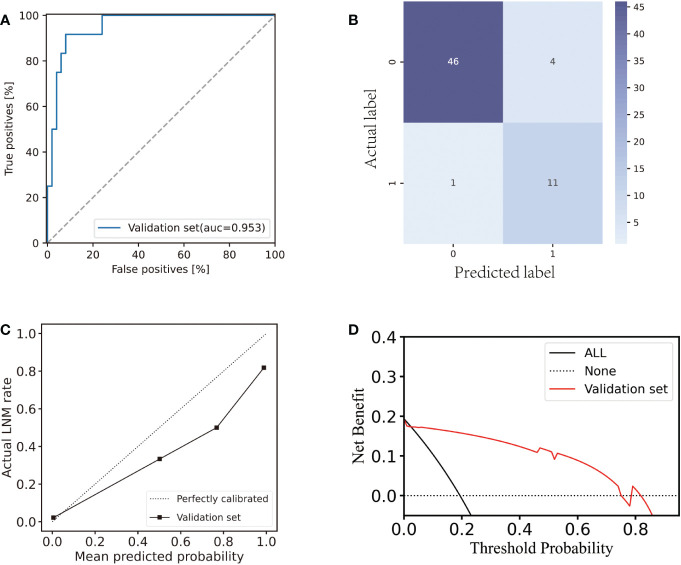
Performance of the AS-based model in the validation set. **(A)** ROC curve of the AS-based model in the validation set. **(B)** Confusion matrix plot for the AS-based model in the validation set. **(C)** Calibration plot for the AS-based model in the validation set. **(D)** Decision curve analysis plot for the AS-based model in the validation set.

Subsequently, the independent validation set was tested with the same LDA classifier. An AUC of 0.953 (**Figure 4A**) was reached by the classifier. The F1-score, specificity, sensitivity, and accuracy were all observed to be 0.815, 0.979, 0.917, and 0.919 respectively. In the validation set, eleven LNM and forty-six non-LNM cases were accurately detected. In the remaining cases, one LNM and four non-LNM cases were incorrectly categorized as non-LNM and LNM, respectively (**Figure 4B**).

In the validation set, there was a strong agreement between the observed LNM rate and the model prediction (**Figure 4C**) revealing good discrimination of the classifier. Hence, our model performed well in the internal testing set. The decision curve analysis (**Figure 4D**) demonstrated that the application of the LDA model to predict LNM in the validation set indicated a greater net benefit increase than the “treat everyone” or “no treatment” strategic scheme over a wide range of threshold probabilities, showing the model’s utility in clinical settings.

### 3.6 Model explanation

**Figure 5:**
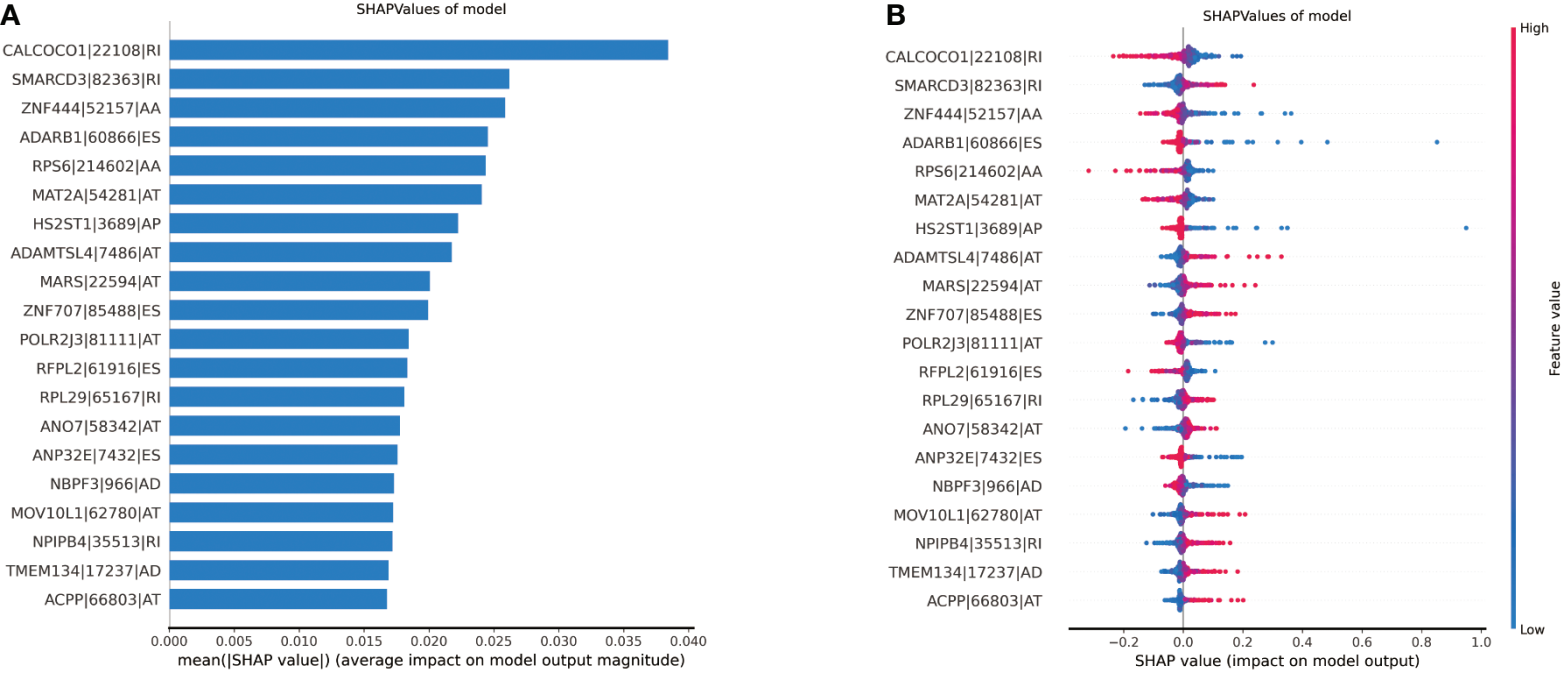
Illustration of the top 20 features contributing to model output by SHAP values. **(A)** The relative contributions of each of the parameters to predict the risk of lymph node metastasis. **(B)** The relative contributions of each feature for model prediction. Features on the right of the risk explanation bar pushed the risk higher, and features on the left pushed the risk lower.

We used SHAP to explain the significance of each feature to the model output. Summary plot was drawn to display the top 20 features, which had the most impact on the model output. **Figures 5A, B** show how high and low the feature values were relative to the SHAP values in the training set. The features were listed from top to bottom in descending order by magnitude of impact on model output, with the first feature having the greatest influence. The parameter values of each feature variable are represented in color on the right side of the variable name, with red representing the high parameter value and blue representing the low parameter value. The higher the SHAP values, the higher the probability of lymph node metastasis. The reverse applies when the SHAP values decrease. The feature with the highest value is CALCOCO1|22108|RI. The lower feature value of CALCOCO1|22108|RI, the higher probability of lymph node metastasis, indicating protecting role of CALCOCO1|22108|RI in LNM.

## 4 Discussion

Precise prediction of LNM in prostate cancer is significant for its prognosis and treatment strategies (20). LNM disease has a worse prognosis and need comprehensive treatment. 50% patients with LNM will suffer clinical and/or biochemical progression within 5 years after radical prostatectomy (21). Androgen deprivation therapy and radiotherapy were currently recommended for LNM disease (20, 22). In recent years, although diagnostic techniques to detect LNM have improved, there is still no highly accurate approach to discriminate between patients with and without lymph node metastases prior to surgery. Extended pelvic lymph node dissection (ePLND) is still the mainstream approach for detecting LNM. The ePLND is not only a diagnostic method of LNM but also a treatment option for LNM. Though, LNM is a small part, many non-LNM patients still have suffered unnecessary ePLND (19, 23).

Recently, the scientific community has increasingly concentrated their efforts on identifying the most trustworthy approaches to predict LNM (19, 24). The AUC of normgrams, imaging modalities, and deep learning models from primary tumor histology ranged from 0.68 to 0.82 (19, 24, 25). Even though, the performance of tools to detect the LNM has been improved, the need for more accurate methods is urgent. High-throughput sequencing has greatly enhanced our ability to gain insight into the root etiology of human disease (26). Genome-wide profiling analysis has been profoundly analyzed in Pca and has contributed to more precise and individualized diagnosis, prevention, and treatment (27–29). Several Genomic-Clinicopathologic nomograms based on RNA_seq have been reported to predict LNM in Pca, gastric cancer, bladder cancer and achieved good performance (30–32). Besides RNA_seq, alternative splicing is another big data from high-throughput sequencing.

Alternative splicing, the process of cleaving the precursor messenger RNA (pre-mRNA), discarding introns and splicing alternative exons, is a crucial procedure in the post-transcriptional gene expression pathway regulation, which leads to multifunctional proteins from a single pre-mRNA (10, 33). AS is extensively involved in many kinds of physiological processes, such as aging, angiogenesis, mammal spermatogenesis, and cornel development (34–37). In addition to physiological processes, AS also plays an important role in tumors. AS changes are constantly observed in many tumors and treated as of a great significance in tumor progression and therapy (38). AS is frequently reported in prostate cancer and plays an important role in prostate cancer progression, castration resistance (39). However, as of now, no single prediction model of lymph node metastasis according to AS data has been reported. Hence, we argued that the types of AS in prostate cancer without lymph node metastasis are different from those in cancer with lymph node metastasis and could identify LNM from non-LNM. In our study, we built a model to predict lymph node metastasis in prostate cancer according to AS signatures.

In our work, the results show that LNM has a particular AS pattern when compared with non-LNM and can distinguish LNM from non-LNM easily. We built a machine learning model to detect LNM only using the AS signatures. The model consisted of 96 AS signatures, with AUC of 0.962 ± 0.026 in the training set and 0.953 in the validation set, respectively. The model also had good sensitivities in the training set and the validation set. Previously, as mentioned above, Zhang et al. built a genomic-clinicopathologic nomogram to predict LNM (30). However, compared to their model, our model has a better performance with an AUC 0.962 vs 0.947 in the training set and 0.953 vs 0.901 in testing test. Furthermore, they used RNA_seq data other than AS data. Radiomics models with machine learning to predict LNM based on MRI or CT were reported in recent years (40, 41). The AUCs of these radiomics models ranged from 0.915 to 0.950, which did not show better performance than our AS model. The DCA analysis shows that our model has good utility in clinical practice. The specific AS signatures can be identified from biopsy specimens before surgery. Hence, our model can facilitate to detect the presence or absence of nodal metastasis at the time of histological diagnosis of Pca. Using the model, many patients without LNM can be spared from ePLND and some patients with LNM can be identified under the circumstances of being undetectable by imaging methods.

We acknowledge the limitations of our study. Our model was performed only in a single institution. It is necessary to be validated in other independent institutions. In addition, since the data of our model was obtained from surgical specimens, further high-throughput sequencing from biopsy specimens is warranted to validate our model.

In summary, we constructed and validated a well-performed AS-based machine learning model that precisely identified lymph node metastasis in Pca. This model enables detection of LNM before surgery, which may optimize integrated tumor diagnosis and treatment in clinical practice and promote tumor prognosis.

## Data availability statement

The original contributions presented in the study are included in the article/**Supplementary Material**. Further inquiries can be directed to the corresponding author.

## Author contributions

PX, JB: Conceptualization, Formal analysis, Writing original draft. XA: Methodology. MY and LJ: Software. XF: Visualization. YL: Writing review and editing. All authors contributed to the article and approved the submitted version.

## Supplementary material

Supplementary Figure 1 Overview of seven types of AS events profiling in the PRAD patients. **(A)** UpSet plot of intersections among seven types of AS events in the PRAD patients. **(B)** Number of AS events and corresponding parent genes in the PRAD patients. **(C)** Pie chart of composition ration of seven AS events in the PRAD patients. **(D)** UpSet plot of intersections among seven types of DEAS events associated with lymph node metastasis in the PRAD patients. **(E)** Number of DEAS events and corresponding parent genes. (**F**) Pie chart of composition ration of seven DEAS events. **(G)** The top 50 DEAS were shown in the heatmap.

Supplementary Table 1 Training set and validation set

Supplementary Table 2 The results of differential analysis of alternative splicing.

Supplementary Table 3 The 96 AS with their corresponding non-zero coefficients from LASSO results.

Supplementary Table 4 The performance of the eight classifiers in the training set with five-fold cross-validation.
