# Supplementary figures and images for: Novel, alternative splicing signature to detect lymph node metastasis in prostate adenocarcinoma with machine learning
Source: Front Oncol. 2023 Jan 13;12:1084403. doi: 10.3389/fonc.2022.1084403 (PMC9880415; doi:10.3389/fonc.2022.1084403)

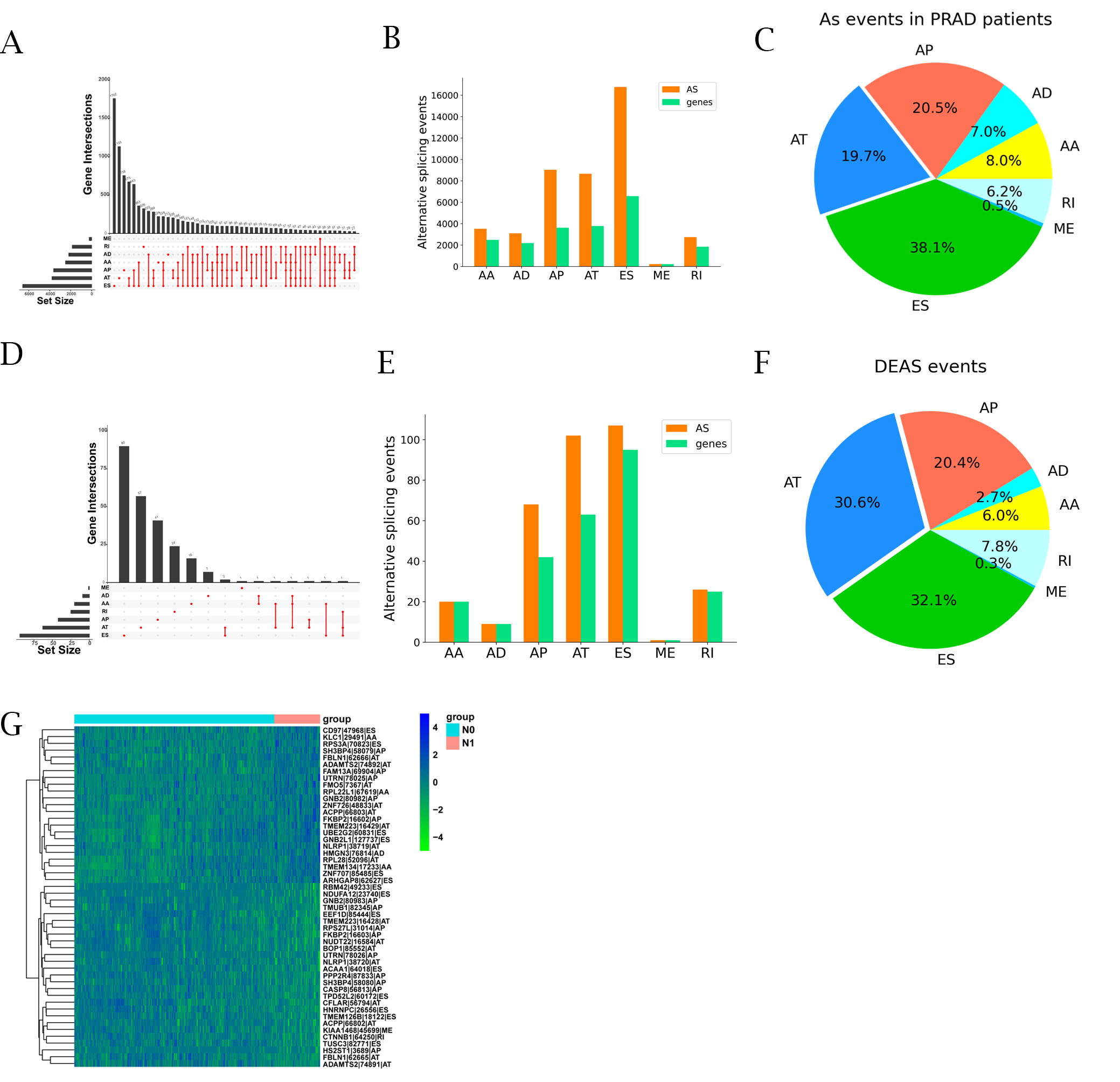

Supplement: Supplementary Figure 1 — Overview of seven types of AS events profiling in the PRAD patients. (A) UpSet plot of intersections among seven types of AS events in the PRAD patients. (B) Number of AS events and corresponding parent genes in the PRAD patients. (C) Pie chart of composition ration of seven AS events in the PRAD patients. (D) UpSet plot of intersections among seven types of DEAS events associated with lymph node metastasis in the PRAD patients. (E) Number of DEAS events and corresponding parent genes. (F) Pie chart of composition ration of seven DEAS events. (G) The top 50 DEAS were shown in the heatmap. [file Image_1.tif]
